# Meta-analysis of corneal endothelial changes after phacoemulsification in diabetic and non-diabetic patients

**DOI:** 10.1186/s12886-023-02924-2

**Published:** 2023-04-24

**Authors:** Yingqin Yang, Hongtao Chai, Zhixiang Ding, Chengye Tang, Yongshun Liang, Yihong Li, Hao Liang

**Affiliations:** 1 Department of Ophtalmology, The First Affiliated Hospital of Guangxi Medical University, NanNing, 530000 China; 2 Department of Gastroenterology, Affiliated Hospital of Guilin Medical University, Ginlin, 541001 China; 3 Department of Ophtalmology, Affiliated Hospital of Guilin Medical University, Ginlin, 541001 China

**Keywords:** Cornea, Phacoemulsification, Diabetic, Meta-analysis

## Abstract

**Background:**

Currently, there is still controversy about the differential changes in corneal endothelium function and morphology after phacoemulsification between Diabetes Mellitus (DM) and non-Diabetes Mellitus (non-DM) patients. In this study, we aimed to evaluate the influence of phacoemulsification on the corneal endothelium in DM and non-DM patients.

**Methods:**

Databases of PubMed, Embase, Web of Science, and the Cochrane Library were searched for studies published between January 1, 2011 and December 25, 2021. The weighted mean difference and 95% confidence interval were used to estimate the outcomes of statistical analyses performed.

**Results:**

Thirteen studies involving 1744 eyes were included in this meta-analysis. No significant difference was observed in the central corneal thickness (CCT), endothelial cell density (ECD), coefficients of variation (CV), or hexagonal cell percentage (HCP) between the DM and non-DM groups (CCT: *P* = 0.91; ECD: *P* = 0.07; CV: *P *= 0.06; HCP: *P* = 0.09) preoperatively. The CCT was significantly thicker in the DM group at 1 month (*P* = 0.003) and 3 months (*P* = 0.0009) postoperatively, and there was no significant difference at 6 months postoperatively (*P* = 0.26) than non-DM group. The CV was significantly higher and HCP was significantly lower in the DM group at 1 month (CV:P < 0.0001, HCP: *P *= 0.002), with no significant difference at 3 months (CV: *P* = 0.09, HCP: *P* = 0.36) and 6 months (CV: *P* = 0.32, HCP: *P* = 0.36) postoperatively than non-DM group. DM patients had lower ECD than non-DM patients at all postoperative time points (1 month, 3 months: *P* < 0.00001, 6 months: *P* < 0.0001).

**Conclusions:**

The influence of phacoemulsification on corneal endothelial damage is greater in diabetic patients. Moreover, the recovery of corneal endothelial function and morphology is delayed in these patients. Clinicians should be more attentive to the corneal health of DM patients when considering phacoemulsification.

**Supplementary Information:**

The online version contains supplementary material available at 10.1186/s12886-023-02924-2.

## Background

The global prevalence of diabetes mellitus (DM) is increasing and predicted to rise to 10.2% by 2030 and 10.9% by 2045 [1]. Poor blood glucose control, as well as advanced age, are the main risk factors for cataract development [2]. Cataracts are the main cause of visual impairment in individuals aged ≥ 50 years worldwide, accounting for approximately 45% of blindness cases [3]. The treatment for cataracts is mainly surgery, the most common surgical method being phacoemulsification combined with intraocular lens implantation. Although phacoemulsification is a well-established method with few complications, there is still a risk of damage to the corneal endothelium during the procedure. DM, in turn, is considered a risk factor for increased corneal endothelial damage after cataract surgery [4]. Corneal endothelial cells (CECs) of regular size and hexagonal shape form neatly arranged monolayers [5]. CECs rely on tight junctions and adherens junctions, Na^+^/K^+^-ATPase pump activity for paracellular fluid and ion transportation, and form an integral barrier function that plays a key role in regulating corneal hydration and maintaining corneal transparency [6, 7].

Clinically, the following four parameters are mainly used to evaluate the health status of the corneal endothelium: central corneal thickness (CCT), endothelial cell density (ECD), coefficients of variation (CV), and hexagonal cell percentage (HCP). The CCT is used as an index to measure corneal endothelial function. The extent of corneal swelling can be estimated by measuring its thickness, and this parameter can be an indicator of the degree of corneal damage that can even cause stromal edema [8]. In humans, where CECs have no regenerative ability, the ECD decreases with age and then tends to be stable. Any damage to CECs is mainly compensated by the expansion and movement of adjacent cells [9]. The CV is an index that reflects the size variability of the endothelial cell area. The HCP refers to the change in the shape of hexagonal cells. The CV and HCP can reflect the repair and healing process occurring upon endothelial cell damage; whenever CECs are damaged, the remaining cells expand and slide, showing an increase in cell size together with a decrease of hexagonal-shaped cells [10].

The health status of the cornea will affect the postoperative recovery of cataract surgery. DM can affect the health of the corneal endothelium [11]. It was suggested that the cornea of diabetic patients is more likely to be damaged after phacoemulsification [4]. In a previous study, researchers systematically analyzed corneal properties early after phacoemulsification (within 3 months) in diabetic and non-diabetic patients [12], although they did not conduct subsequent follow-up studies. Currently, there is still controversy about the long-term differential changes in corneal function and morphology after phacoemulsification between diabetic and non-diabetic patients. In this study, we aimed to evaluate the influence and potential risks of phacoemulsification on the cornea of diabetic and non-diabetic patients by reporting any changes in the CCT, ECD, CV, and HCP within 6 months after phacoemulsification. It is hoped to find the cause of corneal endothelium related complications in diabetic patients after phacoemulsification, which is helpful for clinical treatment.

## Methods

### Inclusion and exclusion criteria

The study included prospective studies. We included patients (1) with and without diabetes who underwent phacoemulsification and intraocular lens implantation, (2) whose outcomes included at least one data index of corneal properties (CCT, ECD, CV, and HCP), (3) with no other systemic diseases except DM, (4) whose blood glucose levels were stable, and (5) with no serious surgery-related complications. Patients with severe ocular and systemic complications caused by DM were excluded, such as those with proliferative diabetic retinopathy (PDR) and diabetic nephropathy. Those with mature cataracts (brown/white), cataract grade V, or other eye diseases were also excluded.

### Search strategy and quality assessment

We selected relevant studies published between January 1, 2011 and December 25, 2021, by searching the databases PubMed, Embase, Web of Science, and the Cochrane Library (Trials Central). No language restrictions were applied. We used the following MeSH terms and Text Words: The complete search used for PubMed was: (((“Cataract”[Mesh]) OR (Cataracts [Title/Abstract])) OR (Lens Opacity* [Title/Abstract])) OR (Opaciti*, Lens [Title/ Abstract])) OR (Cataract*, Membranous [Title/Abstract])) OR (Membranous Cataract* [Title/Abstract])) OR (Pseudoaphakia [Title/Abstract])) OR (Phacoemulsification* [Title/Abstract]))) AND ((“Diabetes Mellitus”[Mesh]) OR (diabete* [Title /Abstract]) OR (diabetic* [Title/ Abstract])) AND ((“Cornea”[Mesh]) OR (Cornea* [Title/ Abstract])). Filters: from 2011/1/1 to 2021/12/25. Manual search was conducted on the reference lists of published key articles in English.

The quality of the selected studies was assessed using the Newcastle–Ottawa Scale (NOS) CASE CONTROL STUDIES, which includes three sections: selection (four items, four points), comparability (one item, two points), and exposure (three items, three points); a total of nine points is achievable, with scores ≥ 6 indicating good quality. Detailed items for the NOS are provided in Additional file 1.

### Data extraction

Two independent investigators extracted the following information: first author and country, publication year, type of study, follow-up duration, patient age, number of eyes, ascertainment criteria for DM and cataracts, DM status (duration or fasting blood sugar or glycated hemoglobin [HbA1c]), presence of diabetic retinopathy, and literature quality assessment scores.

### Statistical analysis

A forest plot was constructed and statistical and sensitivity analyses were performed using Review Manager 5.4.1. Sensitivity analysis was performed using the one-by-one exclusion method. The weighted mean difference (WMD) and 95% confidence interval (CI) were calculated based on selected outcomes. *P* < 0.05 was considered a statistically significant difference. *I*^*2*^ test and Cochran’s *Q* test were used to evaluate heterogeneity. No heterogeneity was indicated by *I*^2^ < 50% and *P* > 0.1, and the fixed-effect model was used to calculate pooled effect. If there was significant heterogeneity, a random effect model was used.

### Publication bias estimate

Stata 14.0 was used for subgroup analysis and the publication bias test. The Egger’s test was used to estimate the publication bias. *P* < 0.05 was considered a statistically significant publication bias. The trim-and-fill method was used to evaluate the influence of publication bias on the interpretation of the results.

## Results

### Study selection

**Fig. 1:**
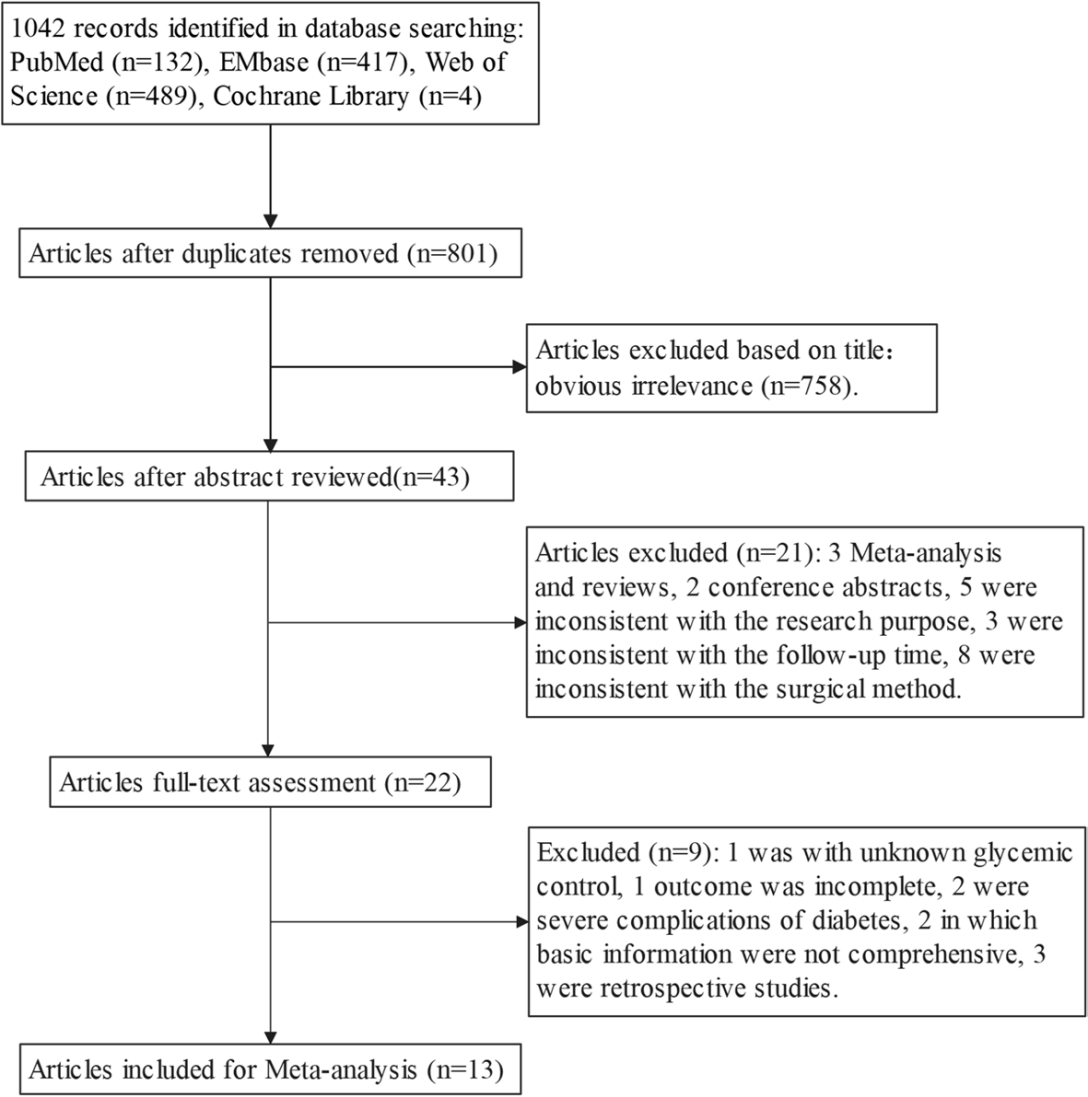
Workflow diagram of literature selection process

The literature selection process is shown in Fig. 1. In total, 1042 relevant studies (PubMed 132, EMbase 417, Web of Science 489, and Cochrane Library 4) were retrieved. Next, they were screened based on redundancy (801 studies remained), screening of topics (43 studies remained), and abstract information (22 studies remained). Nine studies were excluded after reading the full text: one with unknown glycemic control, one with an incomplete outcome index, two in which patients had serious DM complications (PDR surgery history, kidney disease dialysis history), two in which the basic information was not comprehensive, and three retrospective studies. Finally, 13 studies [13–25], including 1744 eyes (788 eyes in the DM group and 956 eyes in the non-DM group), were selected for this meta-analysis.

### Quality assessment of the included literature

According to the NOS, eight studies scored 7, and five studies scored 8. All studies scored more than 6 points, indicating that the quality of the included studies was high. The characteristics of the included studies are provided in Table 1.

**Table 1:** Characteristics of included studies

**Author year**	**Location**	**Type of study**	**Follow up duration(months)**	**Age (year/SD, range)** **_______________**		**No.of eyes** **______________**		**Ascertainment of diabetes**	**Classfication criteria of cataract**	**Diabetes condition**	**DR**	**NOS**
				**DM Group**	**Non-** **DM Group**	**DM Group**	**Non-** **DM Group**					
Hugod [18] 2011	Denmark	Prospective controlled study	3	75.4 ± 9.3	75.6 ± 8.6	30	30	medical history	NA	well control of blood sugar	29 had no DR and 1 had mild NPDR	7
Wang [24] 2013	China	Prospective controlled study	6	65.3 ± 11.0	69.2 ± 8.2	62	82	NA	NA	Fasting blood sugar < 8.0 mmol/L	41 eyes without DR, 21 eyes with DR	7
Zhao [25] 2013	China	Prospective controlled study	3	52 ~ 80	55 ~ 83	56	60	NA	NA	Fasting blood sugar < 8.0 mmol/L	NA	7
Li [13] 2016	China	Prospective controlled study	3	64.58 ± 12.46	65.12 ± 12.30	224	227	Type 2 diabetes clinical diagnostic criteria published by WHO	NA	Duration(y) 1 ~ 3	exclude DR	7
Sahu [23] 2017	India	Prospective controlled study	3	63.38 ± 7.31	64.00 ± 8.32	60	60	American Diabetic Association (ADA 2007)	LOCS III	HbA1c(%) 6.87 ± 0.43	NA	8
Chen [21](1) 2018	China	Prospective controlled study	1	63.56 ± 9.51	62.32 ± 8.37	44	48	NA	NA	Duration(y) 5 ~ 15 ( 8.38 ± 2. 59), HbA1c < 8%	NA	7
Chen [22](2) 2018	China	Prospective controlled study	6	62.8 ± 2.2	63.6 ± 2.4	60	60	NA	NA	Duration(y) 4.4 ± 1.5, well control of blood sugar	No serious diabetic complications	7
Ganesan [15] 2019	India	Prospective controlled study	3	61.0 ± 6.3	58.7 ± 5.5	80	80	NA	NA	well control of blood sugar	NA	7
Khokhar [16] 2019	India	Prospective controlled study	1	58.14 ± 11.96	58.74 ± 11.17	54	194	NA	LOCS III	fasting blood sugar < 140 mg/dL and HbA1c < 7%	No DR or mild NPDR	7
Fernández- Muñoz [19] 2019	Mexico	Prospective controlled study	3	50 ~ 80	50 ~ 80	21	21	Type 2 diabetes clinical diagnostic criteria published by WHO	LOCS II	HbA1c < 6.5% in the previous 5 years	exclude PDR	8
Maadane [20] 2019	Maroc	Prospective controlled study	3	60.42 ± 6.48	62.0 ± 7.21	47	47	American Diabetic Association (ADA 2007)	LOCS III	HbA1c < 7%	NA	8
Budiman [17] 2020	Indonesia	Prospective controlled study	1	60.2 ± 9.4	61.6 ± 12.6	67	86	diabetes history	NA	HbA1c < 10%(7.3 ± 1.08), and/or blood glucose < 200 mg/dL	NA	8
Beato [14] 2021	Portugal	Prospective controlled study	6	72.7 ± 5.7	70.5 ± 6.3	45	43	medical history, HbA1c level ≥ 6.5%, and/or current use of antidiabetic medication	NA	Duration(y) 9.1 ± 8.0; HbA1c levels (%): DM (6.8 ± 1.0), Non-DM (5.5 ± 0.4)	6 eyes with mild to moderate NPDR	8

*DM* Diabetes Mellitus, *HbA1c* Glycosylated Hemoglobin, *DR* Diabetic Retinopathy, *PDR* Proliferative Diabetic Retinopathy, *NPDR* Nonproliferative Diabetic Retinopathy, *NOS* The Newcastle– Ottawa quality assessment scale, *LOCS II* the Lens Opacities Classification System II, *LOCS III* the Lens Opacities Classification System III, *NA* Not Available

### Meta-analysis outcomes

#### CCT

**Fig. 2:**
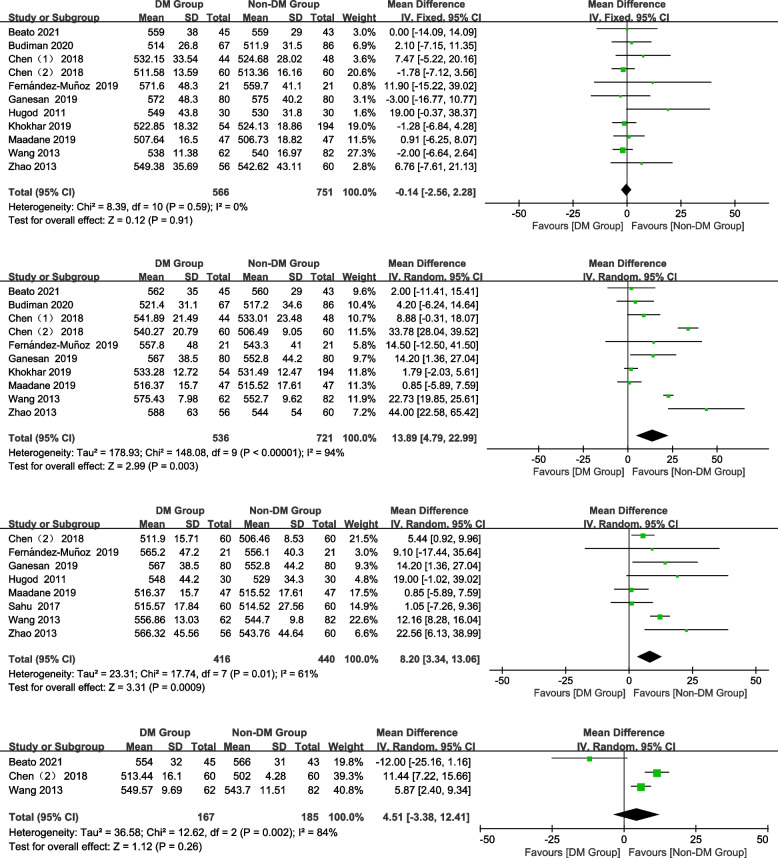
Forest plot of CCT in DM group and non-DM group

In total, 11, 10, 8, and 3 studies were included preoperatively and 1 month, 3 months, and 6 months postoperatively, respectively. No significant difference was observed in CCT between the groups preoperatively and 6 months postoperatively (Fig. 2; preoperative: WMD = -0.14, 95% CI: -2.51–2.28, *Z* = 0.12, *P* = 0.91; 6 months postoperatively: WMD = 4.51, 95% CI: -3.38–12.41, *Z* = 1.12, *P* = 0.26). However, the CCT in the DM group was significantly thicker than that in the non-DM group at 1 month and 3 months postoperatively (Fig. 2; 1 month postoperatively: WMD = 13.89, 95% CI: 4.79–22.99, *Z* = 2.99, *P* = 0.003; 3 months postoperatively: WMD = 8.20, 95% CI: 3.34–13.06, Z = 3.31, *P* = 0.0009).

#### ECD

**Fig. 3:**
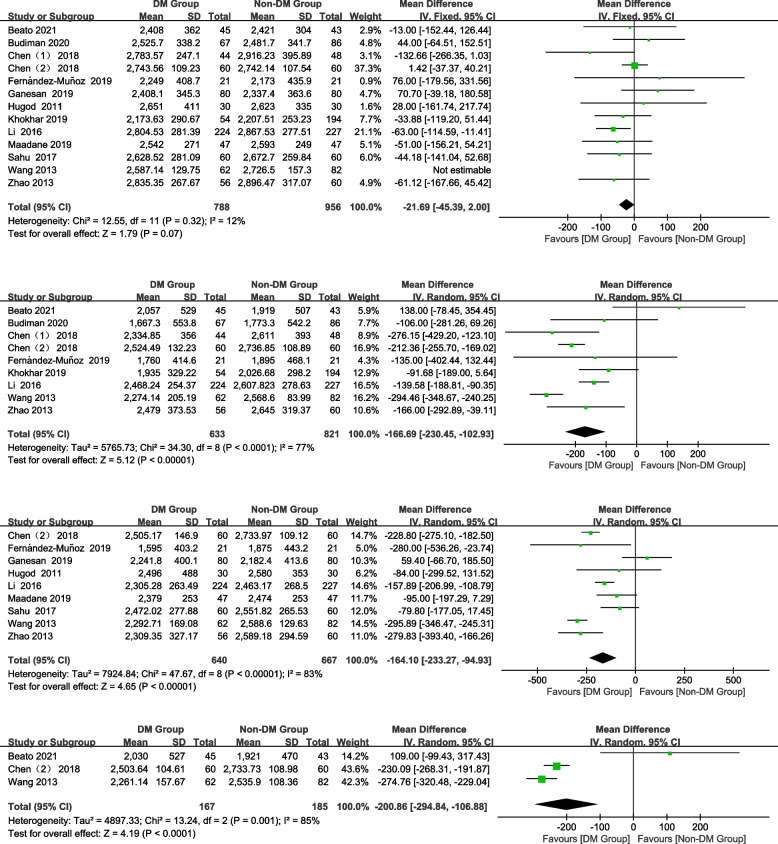
Forest plot of ECD in DM group and non-DM group

In total, 13, 9, 9, and 3 studies were included preoperatively and 1 month, 3 months, and 6 months postoperatively, respectively. There was no significant difference in the ECD between the DM group and non-DM group preoperatively (Fig. 3; WMD = -21.69, 95% CI: -45.39–2.00, *Z* = 1.79,* P* = 0.07). However, patients with DM had a significantly lower ECD than non-DM patients at all postoperative time points (Fig. 3; 1 month postoperatively: WMD = -166.69, 95% CI: -230.45–-102.93, *Z* = 5.12, *P* < 0.00001; 3 months postoperatively: WMD = -164.10, 95% CI:-233.27–-94.93, *Z* = 4.65, *P* < 0.00001; 6 months postoperatively: WMD = -200.86, 95% CI: -294.84–-106.88, *Z* = 4.19, *P* < 0.0001).

#### CV

**Fig. 4:**
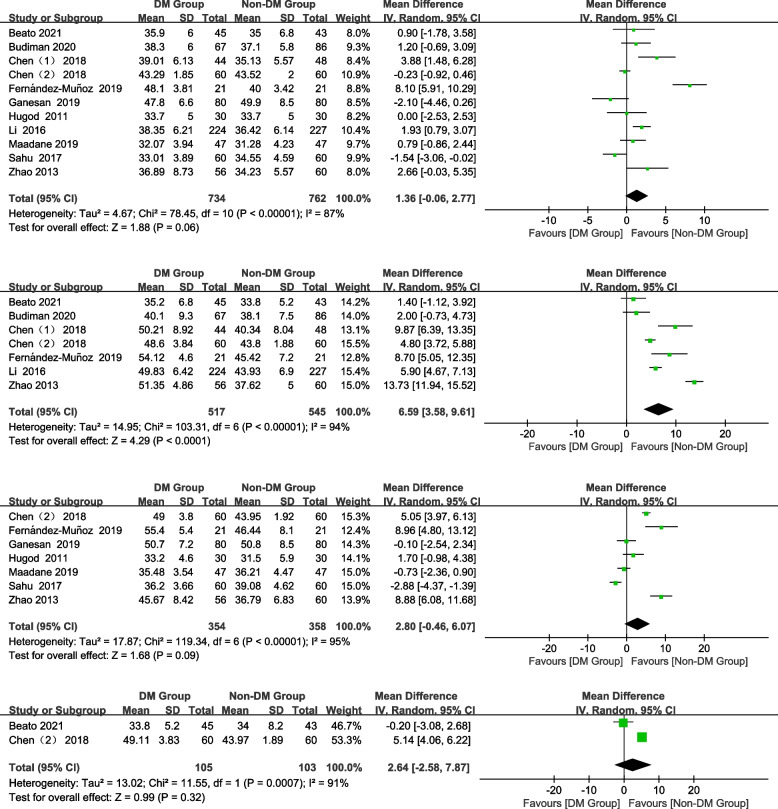
Forest plot of CV in DM group and non-DM group

In total, 11, 7, 7, and 2 studies were included preoperatively and 1 month, 3 months, and 6 months postoperatively, respectively. DM patients had a significantly higher CV at 1 month postoperatively than non-DM patients (Fig. 4; WMD = 6.59, 95% CI: 3.58–9.61, *Z* = 4.29, *P* < 0.0001). No significant difference was found preoperatively and 3 and 6 months postoperatively (Fig. 4; preoperative: WMD = 1.36, 95% CI: -0.06–2.77, *Z* = 1.88, *P* = 0.06; 3 months postoperatively: WMD = 2.80, 95% CI: -0.46–6.07, *Z* = 1.68, *P* = 0.09; 6 months postoperatively: WMD = 2.64, 95% CI: -2.58–7.87, *Z* = 0.99, *P* = 0.32).

#### HCP

**Fig. 5:**
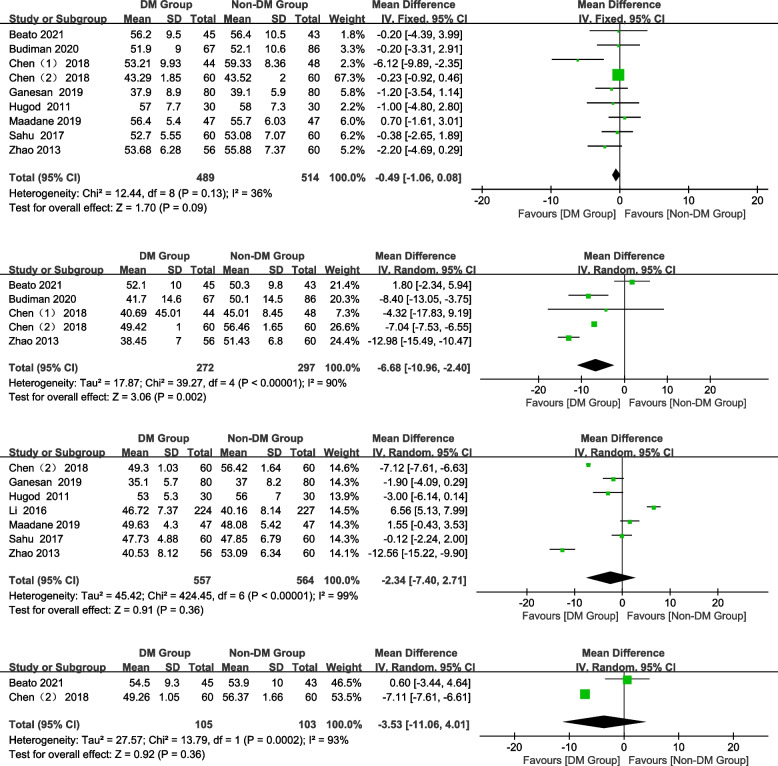
Forest plot of HCP in DM group and non-DM group

In total, 9, 5, 7, and 2 studies were included preoperatively and 1 month, 3 months, and 6 months postoperatively, respectively. The HCP of the DM group was significantly lower than that of the non-DM group at 1 month and postoperatively (Fig. 5; 1 month postoperatively: WMD = -6.68, 95% CI: -10.96–-2.4, *Z* = 3.06, *P* = 0.002). No significant differences were observed in the HCP between the groups preoperatively and at 3, 6 months postoperatively (Fig. 5; preoperative: WMD = -0.49, 95% CI: -1.06–0.08, Z = 1.70, *P* = 0.09; 3 months postoperatively: WMD = -2.34, 95% CI:-7.40–2.71, Z = 0.91, *P* = 0.36; 6 months postoperatively: WMD = -3.53, 95% CI: -11.06–4.01, *Z* = 0.92, *P* = 0.36).

### Sensitivity

Sensitivity analysis publication bias analysis showed that the data of Li [13] were extremely unstable regarding the CV 3 months postoperatively and HCP preoperatively and 1 month and 3 months postoperatively; thus, these data were excluded from this analysis. Although partial results showed relatively large heterogeneity, the data were stable and reliable after sensitivity analysis. Sensitivity analysis was not performed at 6 months postoperatively owing to the small number of included studies.

### Publication bias

The Egger’s test was used to estimate publication bias. Because of the low number of included studies for CV and HCP at 6 months postoperatively, no publication bias analysis was performed at this stage. There was no publication bias in the included studies, except for preoperative CCT (Table 2).

**Table 2:** Publication bias

Time	CCT	ECD	CV	HCP
preoperative	0.012	0.656	0.468	0.662
postoperative 1 month	0.231	0.603	0.392	0.106
postoperative 3 months	0.719	0.577	0.342	0.069
postoperative 6 months	0.579	0.826	NA	NA

*NA* Not Available

**Fig. 6:**
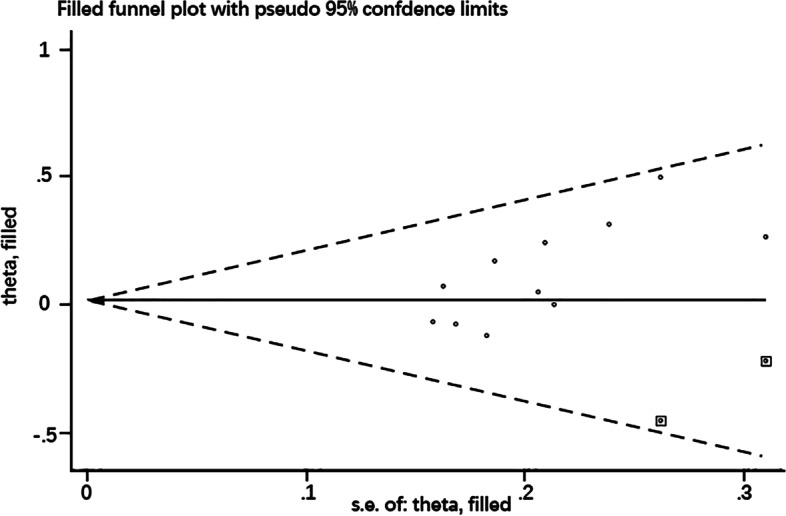
Funnel plot after using the trim-and-fill method

The influence of preoperative CCT publication bias on the interpretation of the results was evaluated using the trim-and-fill method. The pooled effect sizes calculated by the fixed-effect model (pooled effect size: standard error of effect size) were 0.041 and 0.009, and the 95% CI was -0.071 to 0.152 and -0.099 to 0.116 before and after using the trim-and-fill method, respectively. No significant difference was found before and after using the trim-and-fill method (*P* = 0.478, *P* = 0.874, respectively). There was no asymmetry in the funnel plot after supplementing two studies (Supplemental studies are shown as “square” in Fig. 6). This showed that publication bias had little effect on the results, and the results were relatively stable (Fig. 6).

## Discussion

CECs are reportedly lost at a rate of 2.5% per year within 10 years after cataract extraction [26], which is four times the normal physiological loss rate [11]. Patients of advanced age, with a long DM duration and poor blood sugar control, are at greater risk of CECs damage [27]. The mechanism of CEC-enhanced damage caused by DM is still unclear and may be related to the accumulation of advanced glycation end products in the CECs, leading to oxidative stress [28].

Oxidative stress decreases antioxidant levels and increases lipid peroxidation, resulting in CEC damage [29]. Corneal ultrastructural changes, mitochondrial swelling, and impaired function in patients with DM can lead to a decrease in ATP production and pump function in CECs [30]. Importantly, DM also reduces the activity of Na^+^/K^+^-ATP enzymes in endothelial cells [31], which is vital for maintaining endothelial cell function.

The ECD and HCP have been reported to be significantly lower, and the CV and CCT significantly higher in DM patients than in healthy controls [11, 27]. This was even more evident in patients with poor DM status, such as longer diabetes duration (≥ 10 years) and higher HbA1c levels (≥ 7%) [27]. In the present study, there were no significant differences in CCT, ECD, CV, and HCP preoperatively. These findings could be due to the age-specific cataract patients included in our study (50–80 years of age); non-cataract populations of other age groups were not included. Furthermore, we did not perform subgroup analysis on diabetes status (such as disease course and HbA1c level).

Decreased innervation, exposure to vitreous humor [32], increased hardness of lens nucleus [14], surgical trauma [33], intraoperative inflammatory response [15], and postoperative corneal edema [34] are important risk factors for CECs damage after phacoemulsification. However, the risk of CECs injury caused by the above factors increases in diabetic patients. The aim of modern cataract surgery is not only to improve vision but also to minimize the damage to CECs, especially in patients with cataracts and DM.

### CCT

The hydration balance is regulated by the CEC pump in normal conditions. When the CEC pump is dysfunctional, the corneal stroma accumulates water, and swelling occurs, which is manifested by an increase in corneal thickness. However, persistent corneal edema and dysfunction do not occur unless the CEC number declines to < 500–1000 cells/mm [35].

The CCT of patients with DM has been found to be significantly higher than that of healthy individuals, and HbA1c is found to be positively correlated with CCT and CV and negatively correlated with ECD in patients with DM [35, 36]. The duration of DM has a significant impact on these parameters: the longer the DM duration, the higher the CCT and the lower the ECD [37]. In the present study, we found that CCT in the DM group was significantly higher than that in the non-DM group at the early postoperative period (3 months), suggesting that the impairment degree of corneal endothelial barrier function in the DM group was significantly higher than that in the non-DM group. From 3 to 6 months postoperatively, the difference in CCT between the two groups gradually decreased, indicating gradual recovery of the corneal endothelial function. Thus, the corneal endothelial barrier function was impaired at the early postoperative period and then gradually stabilized until 6 months postoperatively. This may be related to postoperative oxidative stress and inflammation response. DM itself [38] and surgical trauma [39] increase the oxidative stress level of CECs. Oxidative stress not only directly damages CECs [29] but also induces inflammation through multiple activation pathways [40]. Corneal edema alleviates with a decrease in inflammation, resulting in a lower CCT during the recovery process after phacoemulsification [15].

### ECD

The percentage of endothelial cell loss (ECL%) in patients with DM was reported to be significantly higher than that in the control group after phacoemulsification [41, 42], and the damage was not restored to the preoperative state at 6 months postoperatively [14]. Joo et al. [43] found that the ECL in patients with DM was higher than that in non-DM patients 1 year after phacoemulsification, although not statistically significant. Furthermore, the duration of DM may affect postoperative ECD loss, with more ECD loss occurring when the duration is ≥ 10 years. Choi et al. [34] found that ECL% was about 2.06 ± 1.36% per year 10 years after phacoemulsification, and this persistent ECL may be related to corneal endothelial remodeling. Ganesan et al. [15] considered inflammation to be a risk factor for ECL in DM patients, whereas age and effective phacoemulsification time were the risk factors in non-DM patients after phacoemulsification.

Our results showed that there was no significant difference in ECD between the DM group and non-DM group preoperatively. However, the ECD in the DM group was significantly lower than that in the non-DM group and the ECL increased progressively compared with that in the non-DM group at 1–6 months postoperatively. This indicated that ECL was accelerated, which was unstable at 6 months postoperatively, and postoperative corneal recovery was delayed in patients with DM. Although the ECL% in patients with DM was higher than that in the control group after phacoemulsification, the intraoperative cumulative dissipated energy (CDE), fluid consumption, and operative time were not statistically significant [41]. The higher ECL postoperatively may be related to the advanced age of patients, increased cataract density, increased endothelial cell vulnerability in diabetic patients, increased trauma during cataract surgery, and grade of cataract [14, 16, 41].

### CV and HCP

CV and HCP reflect the dynamic repair and healing process of CEC morphology after injury; the increase in CV indicates a large variability in cell size, and the decrease in HCP indicates an increase in pleomorphism. The remaining cells expand and slide after endothelial cell injury, which shows an increase in CV and a decrease in HCP. The morphology of CECs in patients with DM was unstable at 4 weeks after phacoemulsification [17]. The HCP of patients with DM decreased significantly 3 months postoperatively, whereas the CV showed no significant difference [18]. However, some studies reported that the CV of patients with DM was significantly higher than that of those without DM at 3 months postoperatively, although this difference did not affect the corneal function [19]. The HCP returned to its preoperative state 6 months postoperatively [14]. No significant change was found in the CV and HCP in either group at 1 year postoperatively [43].

Our results showed that the degree of morphological variation of CECs in diabetic patients was largest at 1 month postoperatively, which was significantly higher than that in non-diabetic patients, and subsequently, the degree of morphological variation of CECs gradually decreased. The corneal morphology of diabetic patients was more unstable in the early postoperative stage, indicating that the endothelial cells of diabetic patients have a weaker repair ability upon damage, and the repair process takes longer [20].

Despite the fact that there was no significant difference in the visual acuity between DM and non-DM patients after phacoemulsification was performed in controlled blood glucose levels [18], the impact of diabetes on corneal health cannot be ignored, as good control of blood glucose is frequently lost in DM. Compared with healthy individuals, the CECs of patients with DM have a lower tolerance to phacoemulsification, are more likely to be damaged, and take longer time to recover, which requires the surgeon to carefully protect the cornea in order to minimize corneal endothelial damage intraoperatively. Femtosecond laser-assisted cataract surgery (FLACS) is reported to cause less damage to the corneal endothelium in patients with DM and can reduce the ECL. This may be because the corneal endothelial injury caused by the small energy during FLACS is insufficient to cause significant damage [44]. Therefore, FLACS may be a better option for patients with DM than conventional phacoemulsification. Furthermore, it should not be ignored that age and DM status are important factors affecting corneal ECD. For DM patients who require cataract surgery, timely surgery is also important when blood glucose is well controlled.

Our study has some limitations. First, intraoperative CDE, fluid consumption, and operative time were not assessed, although most of the included studies showed no statistical differences between the two groups. Second, a randomized controlled trial could not be performed because of DM presence. Third, the cataract grade and expertise of operating surgeon cannot be standardized across all studies, which may be a confounding factor in this study. Finally, longer studies after 6 months, as well as subgroup analyses of DM status (such as DM duration and HbA1c level), were not performed because few studies were eligible for inclusion. In the future, we will continue to focus on the long-term dynamic changes in corneal properties after cataract surgery in patients with DM.

## Conclusion

We conducted a longer dynamic and comprehensive analysis of the changes in corneal function and morphology in DM and non-DM patients after phacoemulsification and evaluated the repair process of corneal injury. Our study showed that the CCT and corneal endothelial morphology were greatly damaged in diabetes patients in the early period after phacoemulsification, but they gradually stabilized during the repair process from 1 to 6 months postoperatively. However, ECD was unstable at 6 months postoperatively in DM patients because the ECL in diabetic patients was still significantly higher than that in non-DM patients. This suggests that more than 6 months are required to recover corneal endothelial function and morphology in DM patients after phacoemulsification. This indicated that DM patients have a higher endothelial loss rate, delayed recovery time, and require a longer follow-up duration after phacoemulsification. Therefore, clinicians should be more attentive to the corneal health of DM patients when considering phacoemulsification.

## Supplementary Information

**Additional file 1.**

## Data Availability

All data generated or analyzed during this study are included in relevant published articles.

## Abbreviations

DM: Diabetes Mellitus
non-DM: Non-Diabetes Mellitus
CCT: Central corneal thickness
ECD: Endothelial cell density
CV: Coefficients of variation
HCP: Hexagonal cell percentage
CECs: Corneal endothelial cells
WMD: Weighted mean difference
CI: Confidence interval
ECL: Endothelial cell loss
CDE: Cumulative dissipated energy
FLACS: Femtosecond laser-assisted cataract surgery
