# Supplementary material for: Meta-analysis of corneal endothelial changes after phacoemulsification in diabetic and non-diabetic patients
Source: BMC Ophthalmol. 2023 Apr 24;23:174. doi: 10.1186/s12886-023-02924-2 (PMC10124059; doi:10.1186/s12886-023-02924-2)
Supplement: Supplementary file 1 — Additional file 1. [file 12886_2023_2924_MOESM1_ESM.pdf]

NEWCASTLE - OTTAWA QUALITY ASSESSMENT SCALE
CASE CONTROL STUDIES

Note: A study can be awarded a maximum of one star for each numbered item within the Selection and Exposure categories. A maximum of two stars can be given for Comparability.

Selection

1) Is the case definition adequate?
   a) yes, with independent validation ✵
   b) yes, eg record linkage or based on self reports
   c) no description

2) Representativeness of the cases
   a) consecutive or obviously representative series of cases ✵
   b) potential for selection biases or not stated

3) Selection of Controls
   a) community controls ✵
   b) hospital controls
   c) no description

4) Definition of Controls
   a) no history of disease (endpoint) ✵
   b) no description of source

Comparability

1) Comparability of cases and controls on the basis of the design or analysis
   a) study controls for _______________ (Select the most important factor.) ✵
   b) study controls for any additional factor ✵ (This criteria could be modified to indicate specific control for a second important factor.)

Exposure

1) Ascertainment of exposure
   a) secure record (eg surgical records) ✵
   b) structured interview where blind to case/control status ✵
   c) interview not blinded to case/control status
   d) written self report or medical record only
   e) no description

2) Same method of ascertainment for cases and controls
   a) yes ✵
   b) no

3) Non-Response rate
   a) same rate for both groups ✵
   b) non respondents described
   c) rate different and no designation
